# Hypoxia in Head and Neck Squamous Cell Carcinoma

**DOI:** 10.5402/2012/708974

**Published:** 2012-10-16

**Authors:** John Zenghong Li, Wei Gao, Jimmy Yu-Wai Chan, Wai-Kuen Ho, Thian-Sze Wong

**Affiliations:** Department of Surgery, Li Ka Shing Faculty of Medicine, The University of Hong Kong, 21 Sassoon Road, Pok Fu Lam, Hong Kong

## Abstract

Hypoxia is a common feature in most of the solid tumors including head and neck squamous cell carcinoma (HNSCC). Hypoxia reflects the imbalance between oxygen consumption by the rapidly proliferating cancer cells and the insufficient oxygen delivery due to poor vascularization and blood supply. The hypoxic microenvironment in the HNSCC contributes to the development of aggressive carcinoma phenotype with high metastatic rate, resistance to therapeutic agents, and higher tumor recurrence rates, leading to low therapeutic efficiency and poor outcome. To overcome the therapeutic resistance due to hypoxia and improving the prognosis of the HNSCC patients, many approaches have been examined in laboratory studies and clinical trials. In this short paper, we discuss the mechanisms involved in the resistance of radiotherapy and chemotherapy in hypoxic condition. We also exploit the molecular mechanisms employed by the HNSCC cells to adapt the hypoxic condition and their tumorigenic role in head and neck, as well as the strategies to overcome hypoxia-induced therapeutic resistance.

## 1. Introduction

Hypoxic microenvironment is frequently found in solid tumors and is known as a negative prognostic factor in head and neck squamous cell carcinoma (HNSCC). Development of hypoxic microenvironment is caused by the imbalance between oxygen consumption and oxygen delivery. The rapidly proliferating HNSCC has insufficient vascularization with poor blood supply. Limiting blood supply network in the rapidly proliferating tumor region limits oxygen diffusion, resulting in the development of hypoxic region. The hypoxic stress stimulates solid tumor to unregulate expression of a variety of oncogenes such as hypoxia-inducible factor and vascular endothelial growth factor, which enhance irregular vascular endothelial cell proliferation and differentiation. The expression of endothelial cell regulators will enhance the growth of new blood capillaries, leading to the development of neovascularized tumors in head and neck [1]. The microvessel network in the solid tumor is physiologically different in comparison with the normal counterpart. Physically, the vasculatures in solid tumors are distended with leaky wall. In terms of efficiency, the blood flow in these newly growth vessels is slow with poor oxygen delivery rate. At present, there is no precise definition of hypoxic oxygen tension in solid tumor as it is highly varying depending on the tumor size and location. The oxygen tension in solid tumor is expressed as partial pressure of oxygen (pO2) with a threshold of 10 mm Hg [2]. In solid tumors, HNSCC is characterized with low oxygen tension. The oxygenation levels in head and neck could be measured at the enlarged cervical lymph nodes and the primary tumor with the use of oxygen-sensitive electrodes and derived from the histography. Becker measured the oxygen tension of primary HNSCC patients and observed that the median tumor pO2 was 8.6 ± 5.4 mm Hg [3]. In advanced HNSCC patients, the measured pO2 was lower. In 67 stage II-III squamous cell carcinoma patients, it was found that pO2 values ≤ 2.5 was a significant prognostic factor for local-regional tumor control and outcome of radiotherapy [4]. Although total tumor volume is a well-known prognostic factor in HNSCC, it is now recognized that the hypoxic volume at the primary site is the key determining factor [5]. Acute hypoxic stress would lead to the development of aggressive cancer phenotype with high metastatic rate, resistance to therapeutic agents, and higher tumor recurrence rates [6–12]. Prolonged deprivation of oxygen will lead to chronic hypoxic stress, leading to tumor necrosis. These features are also observed in HNSCC and now regarded as a major contributing factor leading to the poor outcome [6, 7, 13]. The aim of this short review article is to briefly discuss the mechanisms involved in therapeutic resistance in hypoxic condition. Furthermore, we will exploit the molecular mechanisms employed by the HNSCC cells to adapt the hypoxic condition and their tumorigenic role in head and neck. 

## 2. Hypoxia Contributes to the Poor Therapeutic Outcome in HNSCC

Apart from surgical resection, radiotherapy and chemotherapy are the most common treatment methods in HNSCC patients. The treatment efficacy can be improved by either altered fractionated radiotherapy or concomitant chemoradiotherapy [14]. Accumulated evidence suggested that HNSCC with sufficient oxygen supply has a better responsive rate to radiation in comparison with the hypoxic tumor [15, 16]. Furthermore, oxygen stress could trigger tumor cells to proliferate and allow them to undersurvive cytotoxic-factor assault [17]. 

### 2.1. Hypoxia Contributes to Radioresistance in HNSCC

The tumor cells in the hypoxic region are shown to be more resistant to the radiotherapy compared with well-oxygenated ones [18–20]. It has long been known that the development of hypoxic region in the solid tumor will affect the effect of radiation in killing the cancer cells [21]. Hypoxic radioresistance is first described in 1909. The condition is specific to solid tumor and becomes severe when the oxygen tension of the tumor was 5 mm Hg or less [22]. Quantitative measurement suggested that cells in a hypoxic condition with pO2 of 0.5–20 mm Hg were easier to demonstrate the resistant phenotype [23]. In solid tumor, radiation sensitivity is determined by 2 factors: the intrinsic radiosensitivity of the tumor cells and the degree of hypoxia [24]. Radiation kills the cancer cells by generating reactive hydroxyl free radical with the cellular water. In the presence of oxygen, the reactive free radical will react with the DNA strand, resulting in permanent DNA damage. Oxygen will supply electron to the damaged DNA strand and destabilize the strand break. This enhancement effect of oxygen in radiation therapy is known as oxygen enhancement ratio. Without the electron supply from oxygen under hypoxic condition, the DNA damages induced by radiation could be repaired by the cancer cells. Furthermore, the free radicals generated by the radiation could be neutralized by a sulfhydryl-containing compound [25]. Therefore, in order to reach the same treatment outcome, higher dose of radiation is required in hypoxic cancers in comparison with the anoxic one. In comparison with chronic hypoxia, acute hypoxia was suggested to have a greater contribution to the treatment resistance. This may be due to the higher probability of cell survival and proliferation resuming after oxygen and nutrients deprivation for a limited time [26]. In 1999, Brizel et al. reported that in poorly oxygenated HNSCC patients, 2-year locoregional control, disease-free survival rate, and overall survival rate were significantly lower in comparison with those in the well-oxygenated HNSCC patients [18]. Hypoxia could be developed by smoking. It has been suggested that smoking restricted the radiotherapeutic effect, which might be due to increased level of carboxyhemoglobin and reduced oxygen supply in blood flow of smoking patients [27]. 

### 2.2. Hypoxia Contributes to Chemoresistance in HNSCC

Cancer cell could develop resistance to the chemotherapeutic agents and the hypoxic stress is a promoting factor. Hypoxia-induced chemoresistance was firstly reported in the 1980s, suggesting that tumor cells in the chronically hypoxic or severe hypoxic conditions may be chemoresistant [28, 29]. Sakata et al. also proved the relation between hypoxia and chemoresistance in the mouse breast cell line (EMT6/Ro cells) [30]. In 2001, Matthews et al. treated human breast carcinoma cells and mouse melanoma cells with different chemotherapeutic drugs after exposing the cells to different concentration of oxygen. They found that hypoxia could promote resistance of the tumor cells to the chemotherapeutic drugs including doxorubicin and 5-fluorouracil [31]. In HNSCC, Gabalski et al. observed selective survival of hypoxic tumor cells after chemotherapy [32]. In hypoxic tumor, the chemotherapeutic drugs are less effective. One major reason is the inefficient delivery of the cytotoxic drugs to the tumor sites. As the hypoxic tumor usually has poor vascular structure, diffusion distance of the chemotherapeutic agents to the tumor will be increased, leading to the low delivery rate of the chemotherapeutic drug to the tumor [33–35]. In this circumstance, it is not the tumor itself that develops resistance mechanisms against the applied therapeutic agent. It is the diminished concentration of chemotherapeutic agents in the hypoxic regions resulting in the poor therapeutic efficiency. On the other hand, it was found that the drugs targeting rapidly dividing cells are not useful in eliminating the hypoxic tumors. When the hypoxic region is developed inside the tumor, some hypoxic cells will reduce their proliferation rate and hence reduce the responsiveness to the chemotherapeutic agents [33, 35]. Some of the chemotherapeutic drugs such as carboplatin and doxorubicin were oxygen dependent. These drugs showed low efficiency in the hypoxic condition, also leading to the chemoresistance [34]. Recently, it was discovered that the existence of cancer-derived stem cell is one of the contributing factors leading to the development of drug resistance in HNSCC [36]. In the HNSCC bulk, there is a rare cell population called cancer stem cells. The cancer stem cells in HNSCC have a higher tumor-forming ability in comparison with the anoxia counterpart [37]. The HNSCC stem cells were first reported in 2007 and have the characteristic expression of surface marker CD44 [37]. Apart from CD44, it was later found that HNSCC stem cell would express a subset of stem cell markers including CD133 and aldehyde dehydrogenase [38]. These cancer-derived stem cells have exhibited the ability of self-renewal of normal stem cell and could prevent themselves from entering the cycle of differentiation. In addition, cancer stem cells could survive in fluctuating hypoxic conditions [39]. These cancer stem cells have low proliferation rate and hence are not responsive to the division-dependent therapeutic agents.

## 3. Genetic Factors Involved in Therapeutic Resistance in Hypoxic Condition in HNSCC

Genetic alterations due to hypoxia were associated with radioresistance and chemoresistance in HNSCC. These altered genes and their encoding proteins in hypoxic condition could also serve as the markers for the hypoxia and prognostic prediction. 

### 3.1. Hypoxia-Induced Factors-1 Alpha (HIF-1*α*)

Among all the altered factors involved in hypoxia-induced therapeutic resistance, the hypoxia-induced factors-1 (HIF-1) played a central role. HIF-1 is a heterodimeric basic helix-loop-helix transcription factor composed of two subunits, HIF-1*α* and HIF-1*β*. HIF-1*α* is the central regulatory component, which is firstly described to play a key role in mediating oxygen-dependent transcriptional responses by Wang et al. in 1995 [40]. 

Usually, HIF-1*α* protein only existed in the hypoxic condition, because it was rapidly degraded by ubiquitination under normoxia [40]. Hypoxia suppressed the degradation of HIF-1*α* protein and made it translocate into the tumor cell nuclei from cytoplasm and then form the HIF-1 with HIF-1*β*. After binding to the hypoxia responsive element (HRE) located in the enhancer and promoter regions of its target genes, HIF-1 dysregulated the expression of its downstream genes, which were more than 100 and were reported to be involved in angiogenesis, glucose metabolism, pH regulation, matrix metabolism, proliferation, and apoptosis in the tumors [41]. 

According to the report from Koukourakis et al., HIF-1*α* was not detected in the head and neck mucosa in the noncancerous cases, while it was expressed in the tumor tissues [42]. On the other hand, Costa et al. investigated the hypoxic markers by immunochemical method in 25 adenoid cystic carcinoma cases and they found that HIF-1*α* could be detected in all the tumor tissues [43]. Therefore, HIF-1*α* was thought to be an optimal hypoxic marker and a prognostic predictor for the HNSCC. 

In most studies, HIF-1*α* overexpression was associated with poor outcomes response to the treatment. Aebersold et al. reported their study on the association of HIF-1*α* with the prognosis of the oropharyngeal carcinoma cases after radiotherapy. In this study, 98 cases were processed for immunohistochemistry, and 94% of them showed overexpression of HIF-1*α* in comparison with the normal control [44]. Moreover, higher HIF-1*α* immunoreactivity was related to the lower rate of complete remission of the primary tumor, lymph node metastases, local failure-free survival, disease-free survival, and overall survival. In 2009, Roh et al. reported their study on the prognostic value of hypoxic markers in T2-staged oral tongue cancer. Results suggested that HIF-1*α* overexpression was significantly correlated with the poor disease-specific survival rates [45]. In a study on the radiotherapeutic effect on early-stage glottic carcinoma, HIF-1*α* overexpression was closely correlated with the worse local control of tumor in the early-stage glottis carcinoma after radiotherapy [46].

However, there was contrast result, reported by Beasley et al., showing a positive relationship between HIF-1*α* expression and disease-free and overall survival in the surgically resected patients with or without postoperative radiotherapy. This might because that the effect of reduced proliferation and increased apoptosis induced by HIF-1*α* under hypoxia could overcome the effect of enhancing tumor growth by HIF-1*α* targeted genes in some conditions [47]. Fillies et al. compared the HIF-1*α* expression by immunostaining of early-stage oral floor squamous cell carcinomas, with the tumor size, tumor differentiation, lymph node status, and disease-free and overall survival. They found that low HIF-1*α* expression indicated a poor disease-free and overall survival, while it showed no obvious effect to the tumor size, tumor differentiation, and lymph node status [48]. On the other hand, HIF-1*α* was not necessarily associated with prognosis of the surgically treated supraglottic carcinoma cases, while it was positively correlated with the T-classification of tumor [49]. 

### 3.2. Carbonic Anhydrase IX (CA IX)

Carbonic anhydrase IX (CA IX) is a transmembrane glycoprotein [50], which is firstly thought as a membrane-bound protein “MN protein” on the surface of the HeLa human cervical carcinoma cell line [51, 52]. This protein in the tumor was induced by the hypoxia, while low expression was detected in normoxic tumor region and normal tissues [53–55]. Its encoding gene *CA9* was the downstream gene of HIF-1*α* and PI3K, and its expression was upregulated by increased transcriptional activity of HIF-1*α* and PI3K activation [56, 57]. 

CA IX was reported to be overexpressed in both three HNSCC cell lines under hypoxia and tumor tissues in comparison with the paired normal ones. Moreover, it is located in the perinecrotic area of tumor tissues, where were under hypoxia [50]. It could catalyze the reversible hydration of carbon dioxide to carbonic acid. Its overexpression could reduce the pH value in the extracellular environment, which would alter the uptake and effect of anticancer drugs. With the overexpression of CA IX, the toxicity of weak acidic drugs such as fluorouracil would be enhanced [58] while the toxicity and intracellular accumulation of weak basic drugs such as doxorubicin would be reduced [59].

It was also reported that the carcinoma cells proliferation could be promoted as a result of simultaneous upregulation of proliferative factor-IdUrd and CA IX due to the colocalization of IdUrd and CA IX in HNSCC [60]. CA IX was widely studied as a predictor, usually coupled with other factors, for prognosis of HNSCC patients. In 2001, Koukourakis et al. reported their study with samples of 75 HNSCC patients. Strong cancer cell membrane and cytoplasmic CA IX expression were detected by immunostaining in 26.6% cases, and these cases showed poorer local relapse-free survival and overall survival rate in comparison with the ones with no or low expression of CA IX [61]. Kim et al. reported that the combined high expression of CA IX and Ki 67 indicated a poorer outcome of the patients with squamous cell carcinoma of the tongue. They studied 60 cases and found that the cases with both CA IX and Ki 67 overexpression were related to the lower overall survival rate and shorter disease-free survival period in comparison with the ones with either low expression of CA IX or Ki 67 [62]. In the meta-analysis by Peridis et al., low HIF-1*α*/CAIX expression was significantly correlated with a better prognosis for oral squamous cell carcinoma patients [63]. 

However, some studies showed a reverse relationship between the CA IX and prognosis of HNSCC. According to the study by Eckert et al., oral squamous cell carcinoma patients with higher HIF-1*α* and low CA IX expression showed lower overall survival rate and higher risk of tumor-related death by 4.97-fold in comparison with the patients with low expression of both these proteins [64].

### 3.3. Vascular Endothelial Growth Factor (VEGF)

Vascular endothelial growth factor (VEGF) was at first described as tumor “vascular permeability factor” (VPF) in 1983, which could induce vascular leakage, leading to a high permeability of tumor blood vessels [65]. In 1989, the purified VPF was reported as a regulatory protein for blood vessels growth with modification to the present name [64, 66–68]. In HNSCC, VEGF was reported to be involved in the pathway between tumor hypoxia and neoangiogenesis and as a predictor for the poor prognosis. In a study with 133 HNSCC cases by Dunst et al., tumor oxygenation, tumor hypoxic volume, and serum VEGF level were measured. They found that serum VEGF levels were significantly correlated with hypoxic tumor volume, indicating that the hypoxic condition is the most important influencer to the expression of VEGF [69]. Mohamed et al. also confirmed this result. They studied the relationship between the HIF-1*α* and VEGF on 3 human oral squamous carcinoma cell lines under hypoxia. Significantly higher expression of both HIF-1*α* and VEGF in mRNA and protein level were observed in all the hypoxic oral squamous carcinoma cell lines under hypoxia [1]. In 2007, Liang et al. reported their study on the detection of HIF-1*α* and VEGF expression in 65 cases of oral squamous cell carcinoma. 66.2% and 52.3% of these cases showed HIF-1*α* and VEGF overexpression, respectively. In these cases, higher lymphatic vessel density, regional lymph nodal involvement, and advanced UICC TMN classification were found [70]. Furthermore, the cases with combined overexpression of these two proteins showed a higher blood vessel density. Beside the oral squamous cell carcinoma, this tight correlation between HIF-1*α* and VEGF were also found in laryngeal squamous cell carcinoma [71]. In their study, higher expression of VEGF was also associated with more lymph node involvement and higher recurrence rate. Similar correlation was reported in the nasopharyngeal carcinoma by Hui et al. [72].

## 4. Strategies to Overcome Hypoxia-Induced Therapeutic Resistance 

There are different kinds of strategies such as enhancing oxygen delivery, hypoxic radiosensitizers, and hypoxic cytotoxin to overcome hypoxia-induced therapeutic resistance.

### 4.1. Enhancing Oxygen Delivery

Hyperbaric oxygen (HBO) (100% oxygen) breathing and carbogen (95% oxygen plus 5% carbon dioxide) breathing have been used to elevate the oxygen partial pressure in tissues, thus decreasing chronic hypoxia [73, 74]. HBO therapy refers to inhalation of 100% oxygen at elevated pressure (>1.5 atmospheres absolute), resulting in elevated oxygen level in plasma and tissues [75]. Henk reported that combined 10 fractions of radiotherapy with HBO resulted in higher survival and local control rates in comparison with 30 fractions of radiotherapy in 104 patients with HNSCC, suggesting that HBO augmented the effects of radiotherapy [76]. Ferguson et al. found that HBO therapy could be used as an effective adjunctive treatment modality for laryngeal radionecrosis [77]. In a follow-up clinical study, Haffty et al. evaluated the long-term outcome of treatment with hypofractionated radiation and HBO at 4 atmospheres of pressure in patients with locally advanced laryngeal carcinoma [78]. Clinical data showed that this treatment program resulted in a response rate and long-term tumor control rate comparable to more protracted radiation schedules, indicating that HBO therapy contributed to local control of advanced laryngeal carcinoma [78]. 

Carbogen was suggested as an alternative to pure oxygen because carbon dioxide had vasodilating property [73]. Kaanders et al. carried out a phase II clinical study to assess the effects of ARCON (accelerated radiotherapy with carbogen and nicotinamide) treatment in 215 patients with advanced HNSCC [79]. Nicotinamide was used because it decreased the intermittent closure of blood vessels, leading to the reduction of acute hypoxia [80]. It has been found that ARCON treatment resulted in the local control rates of 80% for larynx, 60% for hypopharynx, 87% for oropharynx, and 29% for oral cavity in patients with T3 and T4 tumors. ARCON treatment also led to the regional control rates of 100% for N0, 93% for N1, and 74% for N2 disease [80]. These results indicated that ARCON treatment exhibited high local and regional control rates in patients with advanced HNSCC.

### 4.2. Hypoxic Cell Radiosensitizers

Two kinds of hypoxic cell radiosensitizer, misonidazole and nimorazole, have been reported [79, 81]. These two compounds mimic the effects of oxygen due to their electron affinity, resulting in the increase in DNA damage and restoration of radiosensitivity. Overgaard et al. assessed the efficacy of misonidazole in 626 patients with pharynx and larynx carcinoma [79]. It has been reported that patients with pharynx carcinomas treated with misonidazole exhibited a significantly better control rate than patients treated with another hypoxic cell radiosensitizer, placebo. However, the clinical use of misonidazole was limited because it caused significant peripheral neuropathy in 26% of the patients [79]. 

Cottrill et al. evaluated the outcome of combined nimorazole with continuous hyperfractionated accelerated radiation therapy (CHART) in 22 patients with locally advanced stage IV HNSCC. Clinical data showed that combined treatment led to higher tumor responses in comparison with CHART regimen alone [82]. Moreover, nimorazole did not enhance the severity of acute normal tissue radiation effects [82]. Subsequently, the same research group carried out a phase II clinical study with 61 patients with advanced stage III or IV HNSCC [83]. Combination of nimorazole and CHART caused the same normal tissue effects as CHART. Furthermore, combination of CHART and nimorazole resulted in the higher local-regional control rates in comparison with CHART alone, suggesting that nimorazole displayed inhibitory effects on HNSCC [83]. In a randomized double-blind phase III clinical study with 422 patients with pharynx and supraglottic larynx carcinoma, Overgaard et al. assessed the efficacy and tolerance of nimorazole in combination with primary radiotherapy [81]. Clinical data showed that patients treated with nimorazole displayed a better locoregional control rate, endpoints of final locoregional control (including surgical salvage), and overall survival than patients treated by another hypoxic cell radiosensitizer, placebo [81]. Furthermore, administration of nimorazole had no major side effects to patients [81]. These results indicated that nimorazole enhanced the inhibitory effects of radiotherapy in patients with pharynx and supraglottic larynx carcinoma.

### 4.3. Hypoxic Cytotoxin

Tirapazamine, a hypoxic cell cytotoxin, displayed cytotoxicity in hypoxia cells but not in normal cells [74]. When exposed in hypoxia conditions, tirapazamine was reduced to a reactive radical, which could lead to single- and double-strand DNA breaks. In contrast, this reactive radical was oxidized to the inert parent compound in normoxia [74]. Rischin et al. performed a clinical study to assess the effects of tirapazamine in combination with cisplatin and radiation in patients with advanced HNSCC [84]. Results from ^18^F misonidazole scans demonstrated that hypoxia levels decreased in patients after treatment. Clinical data also showed that combined tirapazamine with cisplatin and radiotherapy led to good and durable clinical responses with a 3-year failure-free survival rate of 69%, a 3-year local progression-free rate of 88%, and a 3-year overall survival rate of 69% [84]. In a randomized phase II clinical trial carried out by Rischin et al. to select therapeutic regimens for locally advanced HNSCC, it has been shown that patients treated with tirapazamine/cisplatin/radiation displayed higher three-year failure-free survival rates and three-year locoregional failure-free rates than patients treated with cisplatin/fluorouracil/radiation [85]. Furthermore, acute skin radiation reaction was less severe and prolonged in tirapazamine/cisplatin/radiation group than that in cisplatin/fluorouracil/radiation group [85]. Cohen et al. have also found that combined radiation with tirapazamine and cisplatin resulted in long-term survival in a significant proportion of patients [86].

## 5. Conclusion

Hypoxia is a common feature in HNSCC, which contributes to the development of tumorous aggression and metastasis. What's more, it is reported to play a key role in the radioresistance, the chemoresistance, and then the poor prognosis. In view of that, the hypoxic condition should be an important target in the HNSCC treatment. Removing the hypoxic microenvironment by enhancing the oxygen delivery, as well as improving the hypoxic cell death rate by using hypoxic cell radiosensitizer and hypoxic cell cytotoxin, are proved to be able to help to overcome the poor prognosis of HNSCC. On the other hand, since HIF-1*α* and its downstream genes are proved to play central roles in hypoxia-induced therapeutic resistance in HNSCC, exploration for agents targeting to these genes could be another orientation in improving the therapeutic effect and the poor prognosis of HNSCC.
